# Effects of TiO_2_ Nanoparticles Incorporation into Cells of Tomato Roots

**DOI:** 10.3390/nano11051127

**Published:** 2021-04-27

**Authors:** Dulce Estefanía Nicolás-Álvarez, José Alberto Andraca-Adame, José Jorge Chanona-Pérez, Juan Vicente Méndez-Méndez, Raúl Borja-Urby, Nicolás Cayetano-Castro, Hugo Martínez-Gutiérrez, Primavera López-Salazar

**Affiliations:** 1 Departamento de Fisiología, ENCB, Instituto Politécnico Nacional, Av. Wilfrido Massieu Esq. Cda. Miguel Stampa s/n, Gustavo A. Madero, Ciudad de México 07738, Mexico; nialdulceestefania@gmail.com; 2 UPIIH, Instituto Politécnico Nacional, Carretera “Pachuca-Actopan” Kilómetro 1 + 500, Municipio San Agustín Tlaxiaca, Hidalgo 42162, Mexico; 3 Departamento de Ingeniería Bioquímica, ENCB, Instituto Politécnico Nacional, Av. Wilfrido Massieu Esq. Cda. Miguel Stampa s/n, Gustavo A. Madero, Ciudad de México 07738, Mexico; jorge_chanona@hotmail.com; 4 CNMN, Instituto Politécnico Nacional, Wilfrido Massieu s/n, UPALM, Gustavo A. Madero, Ciudad de México 07738, Mexico; jmendezm@ipn.mx (J.V.M.-M.); r_borja_u@hotmail.com (R.B.-U.); nicolas_cayetano@yahoo.com (N.C.-C.); hmartinez63@hotmail.com (H.M.-G.); 5 CIDS, Instituto de Ciencias, Benemérita Universidad Autónoma de Puebla, Av. 14 Sur y San Claudio. Edif. IC6, Ciudad Universitaria, Puebla 72570, Mexico; primaveraster@gmail.com

**Keywords:** TiO_2_ nanoparticles, tomato root, Young’s Modulus, morphology and mechanical properties in cells

## Abstract

In this study, tomato plants were grown in vitro with and without incorporation of TiO_2_ nanoparticles in Murashige and Skoog (MS) growth medium. The aim of this study was to describe the morphological (area and roundness cell) and mechanical (Young’s Modulus) change in the different tissue of tomato root, epidermis (Ep), parenchyma (Pa), and vascular bundles (Vb), when the whole plant was exposed to TiO_2_ nanoparticles (TiO_2_ NPs). light microscopy (LM), scanning electron microscopy (SEM), transmission electron microscopy (TEM), and atomic force microscopy (AFM), wavelength dispersive X-ray fluorescence (WDXRF) techniques were used to identify changes into the root cells when TiO_2_ NPs were incorporated. TiO_2_ NPs incorporation produces changes in the area, roundness, and Young’s Modulus of the tomato root. When tomato root is exposed to TiO_2_ NPs, the Ep and Vb area size decreases from 260.92 µm^2^ to 160.71 µm^2^ and, 103.08 µm^2^ to 52.13 µm^2^, respectively, compared with the control area, while in Pa tissue the area size was increased considerably from 337.72 mm^2^ to 892.96 mm^2^. Cellular roundness was evident in tomato root that was exposed to TiO_2_ NPs in the Ep (0.49 to 0.67), Pa (0.63 to 0.79), and Vb (0.76 to 0.71) area zones. Young’s Modulus in Pa zone showed a rigid mechanical behavior when tomato root is exposed to TiO_2_ NPs (0.48 to 4.98 MPa control and TiO_2_ NPs, respectively). Meanwhile, Ep and Vb were softer than the control sample (13.9 to 1.06 MPa and 6.37 to 4.41 MPa respectively). This means that the Pa zone was stiffer than Ep and Vb when the root is exposed to TiO_2_ NPs. Furthermore, TiO_2_ NPs were internalized in the root tissue of tomato, accumulating mainly in the cell wall and intercellular spaces, with a wide distribution throughout the tissue, as seen in TEM.

## 1. Introduction

TiO_2_ NPs are one of the most abundant nanomaterials produced in the world. They have multiple commercial applications: sunscreens, makeup, various plastic-based products, containers, clothing, catalysts in household cleaning products, self-cleaning coatings, air filtration devices, electronics, hair styling devices, and environmental remediation of pollutants [1]. Emerging applications include solar cells that use TiO_2_ NPs for their electron transfer properties [2]. In addition, food-grade TiO_2_ NPs are also found in a wide variety of foods, such as doughnuts, gum, and candy. Similarly, TiO_2_ NPs are regularly found in personal care products such as toothpaste, sunscreens, shaving creams, shampoo, conditioners, and deodorants [3].

TiO_2_ NPs have many physiological effects in plants, it depends on the particle size, crystallographic phase, concentration, kind of exposure to the plant, the medium growth employed, the species tested, and the exposition time to the nanomaterial. The results could be positive or negative to the plant development [4].

Experiments on plant tissues with TiO_2_ NPs are unclear and sometimes have contradictory results. In some biological systems, they show significant positive effects on seed growth and no statistical difference in development, transpiration rate, and efficient use of water in the seedling [5]. In another plant system, such as Vicia narbonensis (Fabacea) and Zea mays (Poaceae), the TiO_2_ NPs delay the germination process, affecting cell division, and inducing genotoxic effects [6]. In plants of Nicotiana tabacum (Solanaceae) and Allium cepa (Amaryllidaceae), genotoxic effects, and DNA damages were observed, as well as the increase in lipid peroxidation and oxidative stress [7]. Likewise, the effect of TiO_2_ NPs on the physiology of Asian beans (Vigna radiata) has been published; the author reports a beneficial effect and proposes its use as fertilizer [8].

In recent years, the effect of TiO_2_ NPs a handful of plants, such as rice [9,10], Lactuca sativa (lettuce), and Ocimum basilicum (basil), barley [11], and wheat [12,13,14] has been reported. A comparative study of the effects of TiO_2_ and Ag NPs in tomato plants (Lycopersicum esculentum) in order to measure their toxicity was reported in Ref. [5]. Results presented indicate no significant differences in the rate of germination, elongation of the root system, and chlorophyll levels. However, statistical differences in the level of superoxide activity dismutase and total antioxidant capacity were found. 

Other studies show the effect of TiO_2_ NPs in tomato plants with a physiological approach, and hydroponic growth medium. The authors described changes in photosynthesis rate and catalase and peroxidase activity. They show at high concentrations of TiO_2_ NPs (0.5–2 g/L) that the photosynthesis rate increases to 50%, and in high concentration (4 g/L) this parameter is affected negatively [15]. In 2015, Raliya et al. [16] tested TiO_2_ and ZnO nanoparticles of similar size (25 nm) in different concentrations (0 to 1000 mg/kg) in leaf, root and shoot, they found changes in chlorophyll content, becoming higher in plants with exposure to TiO_2_ NPs and have evidence of translocation of TiO_2_ NPs inside the leaf of tomato plant by foliar application in spray TiO_2_ NPs, and soil entrance by a mix with the soil medium. On the other hand, physicochemical alterations in tomato root have been reported when the tomato plant was exposed to strong irradiance and TiO_2_ NPs, that NPs induced several changes in a phenotypic and physiological way, including the increase in fruit and flower production, such as anthocyanin and carotenoids [17].

Thereby, the tomato plant is considered a model to test nanomaterial effects owing to its capability to respond to changes in its growth medium. Some studies exposed tomato plants (root, leaves and fruits) to nanomaterials and found genetic and physiological changes dependent of the physic-chemical properties of nanomaterials, such as Khodakovskaya et al. (2010) [18].

Despite the extensive applications, there are only a few reports that have studied the effects of TiO_2_ NPs interactions with plant species in order to define the stiffness of the cells caused by the exposition to NPs [19,20,21].

In this study, the main objective was to compare the morphological changes (area and roundness cell) and cell stiffness in the different zone in absorption tomato root when the plant is exposed to TiO_2_ NPs in the growth medium and detect the absorption and ubication of this nanomaterial into the root cell. 

## 2. Materials and Methods

TiO_2_ NPs were obtained from Sigma Aldrich, (637254, US). The TiO_2_ NPs are white powders with a size smaller than 25 nm, according to the supplier. The nanoparticles were thoroughly characterized with X-ray diffraction (XRD), dynamic light scattering (DLS), WDXRF, SEM and TEM before being incorporated into MS growth medium for tomato seeds. 

### 2.1. TiO_2_ NPs Characterization Techniques

#### 2.1.1. XRD

TiO_2_ NPs powder was measured with XRD to evaluate the crystalline structure with a MiniFlex 600 from Rigaku (Tokyo, Japan) diffractometer. The sample was placed in a zero-background holder for analysis. Measurements were carried out with Cu Kα (*λ* = 1.5418 Å) radiation as the source with linear focus and operated at 40 kV and 15 mA. The database PDF2–2003 was employed as the reference JCPDS to identify phases in the X-ray diffraction pattern (XRDP). The HighScore Plus® program from PANalytical was used for quantitative analysis for XRDP. The Scherrer Equation (1) was used to determine the crystallite size average [22]:

(1) $$d=\frac{K\lambda }{\beta cos\theta }$$

where: *d* is the crystallite size average, *K* is the shape factor (0.89 for spherical crystalline solids with cubic unit cells), *λ* is the Cu X-ray radiation wavelength (1.542 Å), *θ* is the Bragg angle, and *β* is the full width at half maximum (FWHM) of the XRD without instrumental width.

#### 2.1.2. DLS

TiO_2_ NPs are white powders that tend to agglomerate. To obtain the adequate size distribution, DLS was used with Zetasizer NANO from Malvern, ZSP (New York, NY, USA). TiO_2_ NPs were dispersed in an ethylene glycol-water mix (1:3) sonicated for 20 min before DLS measurements.

#### 2.1.3. WDXRF 

A WDXRF was used for elemental analysis and to determine the percentage of Ti incorporated into the tomato roots. TiO_2_ NPs powders were compressed to form a tablet. The analysis was performed with a QUANT-EXPRESS (fundamental parameters) method in the range of Na to U in a sequential X-ray fluorescence spectrophotometer on the wavelength of 1 kW (Tiger S8, Bruker) with an X-ray source of Rhodium (Rh). 

#### 2.1.4. SEM and TEM

SEM images were obtained with the CCD camera of an electron microscope (JEOL, JSM 7800F, Tokyo, Japan) at 20 kV under high vacuum. To obtain the TEM data, the TiO_2_ NPs were dispersed in isopropyl alcohol and sonicated for 20 min before being dropped on a Cu grid. Digital images were obtained with TEM from JEOL, JEM-ARM, Tokyo, Japan operating at 180 keV under high vacuum.

### 2.2. Plant Culture

#### 2.2.1. Tomato Seeds 

Tomato (*Solanum lycopersicum*) seeds were acquired from the “El semillero” shop in Mexico City. Seeds were immersed in 10% sodium hypochlorite solution for 5 min and then rinsed three times with deionized water to ensure surface sterility. Then the seeds were put into a magenta vessel with MS medium (Sigma Aldrich, M5519, St. Louis, MO, USA), with and without 20 mg/L of TiO_2_ NPs respectively. All experiments were triplicated, and 60 plants were grown and used in this study.

TiO_2_ NPs were suspended directly in deionized water and dispersed by ultrasonic vibration (100 W, 60 kHz) for 30 min. The TiO_2_ NPs were sterilized, added to the MS media at either 0 or 20 mg/L, mixed and put into magenta vessels for the tomato plant culture. 

An environmental chamber (LAB-LINE Biotronette mark III, Burlington, VT, USA) was employed to cultivate the tomato plants in a photoperiod of 12/8 h light/dark at 24 °C, with a relative humidity of 70 ± 25%. The absorption root zone was used for experimental conditions at 21 days of growth. 

#### 2.2.2. LM for Tomato Root Microstructure

The microstructure of tomato root was characterized by LM. Primary root, specifically the absorption zone segments, approximately 1 cm below the stem of tomato root tissue, on day 21 of growth were used to identify the different tissues by LM. LM images were acquired with a 10× objective in RGB and saved in TIFF format at 598 × 598 pixels. Five fields of each root region were used to perform the image analysis.

The area of interest was selected, and it was cut into phosphate buffer and fixed with glutaraldehyde 3% (24 h), then the root passed to alcoholic dehydration with an increase in alcohol concentration from 30 to 100% by steps of 10%. After, the samples were exposed to oxide propylene and a mix of oxide propylene with resin in 3:1, 2:1, 1:3, and resin proportions were substituted for the alcohol in the sample with epoxy resin. The resin blocks were cut in an ultramicrotome, and the sections were stained and observed in LM.

The nanoindentation tests were carried out according to Cardenas-Pérez, et al. (2016) [19]. Sections of approximately 70 nm of thickness were cut using a semiautomatic ultramicrotome (UC7, Leica, Wetzlar, Germany). The slices were stained with safranin and fast green (control) and toluidine blue (1%) (tested root), Safranin stained the Vb and fast green to identify the Pa and Ep tissue. Ep was considered the first layer of cells and observed using an optical microscope (Eclipse Ni-U, Nikon (Tokyo, Japan)). The size and shape of cells in the root tissue were characterized by image analysis; considering an area in mm^2^ and the shape taking values from 0 to 1, where 1 corresponds to a perfectly spherical shape of the cell. LM images were acquired with a 10 X objective in RGB and saved in TIFF format at 598 × 598 pixels. Five fields of each root region were used to perform the image analysis.

### 2.3. TiO_2_ NPs Detection inside Tomato Root

#### 2.3.1. TiO_2_ NPs Detection by WDXRF

Tomato roots with and without TiO_2_ NPs were dried for 48 h in a Red Line oven by BINDER at 60 °C. Each sample was weighed and powdered in an Agatha mortar. The samples were combined with a non-fluorescent powder to compress the sample into a tablet. The same equipment and method used in Section 2.1.3 were employed here for the TiO_2_ NPs characterization at 0 and 20 mg/L concentrations.

#### 2.3.2. Scanning and Transmission Electron Microscopy (SEM and TEM)

Segments of the tomato roots with and without TiO_2_ NPs after 21 days of growth were cut from the absorption zone and fixed with 3% glutaraldehyde over 2 h at room temperature and then fixed again with osmium tetraoxide (OsO_4_) for 1 h. Then, alcoholic dehydration was performed through an increasing series of ethanol percentage from 30% to 40, 50, 60, 70, 80 and 90%, for 20 min. After dehydration, the sample was put into absolute ethanol for 10 min. Alcohol was decanted, and propylene oxide was added for 20 min. Next, the sample was put in a mixture of propylene oxide and resin in proportions of 2:1, 1:1, 1:3 and 1:0, for 24 h per mix. Finally, the samples were carefully mounted in a silicone mold and polymerized at 60 °C for 48 h.

Polymerized samples were obtained in ultrathin slices of 70 nm with an ultramicrotome (EM UC7, LEICA) at environmental conditions. The slices were put on a grid of 70 mesh with formvar and contrasted with uranyl acetate and lead citrate for 5 min each one. The analysis was done in a TEM (JEM-2100) at 80 kV.

For SEM measurements, the absorption zone segment of the tomato root was fixed and dehydrated in the alcohol series. Like the method described previously, the samples were processed to a critical point of drying with ultra-dry CO_2_ (K850, Quorum, UK) and mounted in an aluminum sample port with carbon tape. The samples were not covered in gold or another conductive material to avoid altering nanoparticle composition with the covering material. In contrast to the commonly applied conductive layer deposition on the SEM simple surfaces, these simple were left uncovered to avoid contamination. The samples were analyzed by energy dispersive X-ray spectroscopy (EDS).

### 2.4. Nanomechanical Properties

#### 2.4.1. AFM

The indentation tests were performed in liquid media (MS at 1% *w*/*v*) with AFM perfusion cell. The “Point and shoot” method was employed to measure Young’s Modulus (*E*) according to Cárdenas-Pérez et al. [15]. *E* was obtained by a nanoindentation technique from each force-curve calculated using the Sneddon model. AFM conditions (Bruker, Bioscope Catalyst ScanAsyst, Camarillo, MA, USA) and a Poisson ratio of 0.3 for soft biological samples were considered.

NP-10 probes tips (Bruker) with V-shaped cantilever and 20 nm radius pyramidal-geometry were used to indent the samples. The thermal tune cantilever calibration method was repeated three times, and an average k range between 0.49 ± 0.04 N/m to 0.53 ± 0.02 N/m was obtained. Calibration was performed with the hard surface of a glass slide. The indentation (δ) of this material was assigned 0 because it is a hard surface, and the piezoelectric movement (z) corresponds to cantilever deflection.

#### 2.4.2. Nanoindentation and Image Data Analysis

Each treatment was performed in triplicate, and the results were expressed with standard error bars. Statistical differences of the experimental data were examined by the Student *t*-test correspondent control. All the statistical analysis were implemented using SigmaPlot v. 12.0 (SYSTAT, Inc., San Jose, CA, USA). A significant difference was defined as that with a *p* value < 0.05. 

## 3. Results

### 3.1. TiO_2_ NPs Characterization

Since TiO_2_ NPs properties are different in agglomerated and monodispersed forms. TiO_2_ NPs were characterized to determine their initial state. TiO_2_ NPs phases and crystalline size average were determined by XRD. For TiO_2_ NPs size distributions, DLS is performed, and for shape TEM and SEM were used. Then, TiO_2_ NPs were dispersed and incorporated into MS growth medium for growth tomato seeds. 

**Figure 1:**
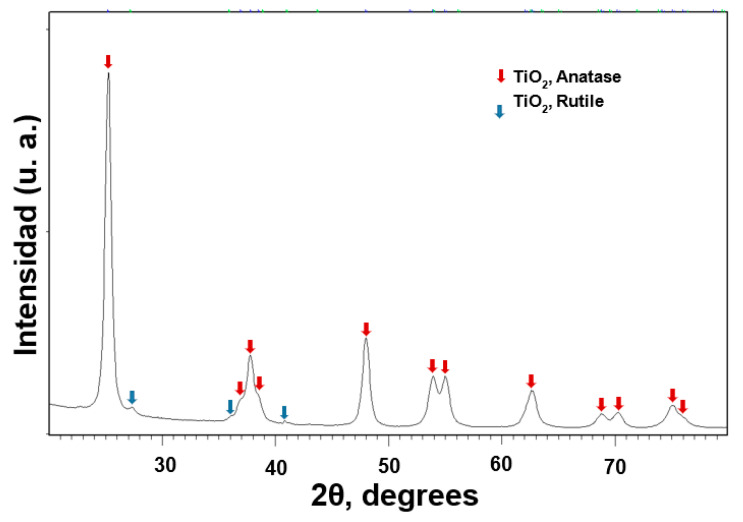
X-ray Diffraction Pattern from TiO_2_ NPs.

Figure 1 shows the X-ray Diffraction Pattern (XRDP) from TiO_2_ NPs. The XRDP was compared with diffraction pattens from ICDD (PDF 2003) database, and the peaks were found to match with the TiO_2_ anatase phase (98-002-4276). Only a small peak is observed (27.43°) corresponding to the Rutile phase (980009161). The quantitative analysis with the Rietveld method obtained a percentage of 97.4% and 2.6% for anatase and rutile, respectively. Furthermore, the average crystal size of 14 ± 1 nm was determined according to the Scherrer equation (Equation (1)) from 25.28° peak.

**Figure 2:**
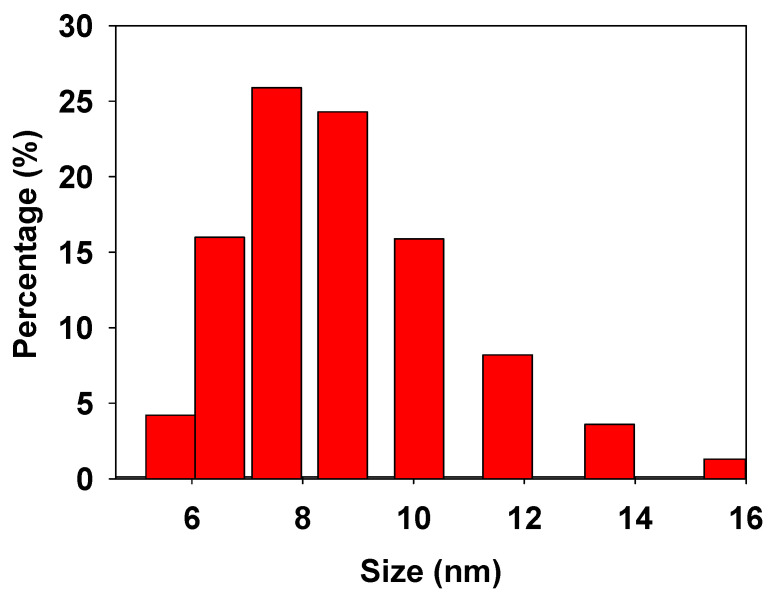
Size distribution of TiO_2_ NPs determined by DLS.

Figure 2 shows the DLS result of the size distribution of TiO_2_ NPs. The NPs have a hydrodynamic diameter size distribution, expressed as number percentage, around 7.5 ± 2.1 nm.

**Figure 3:**
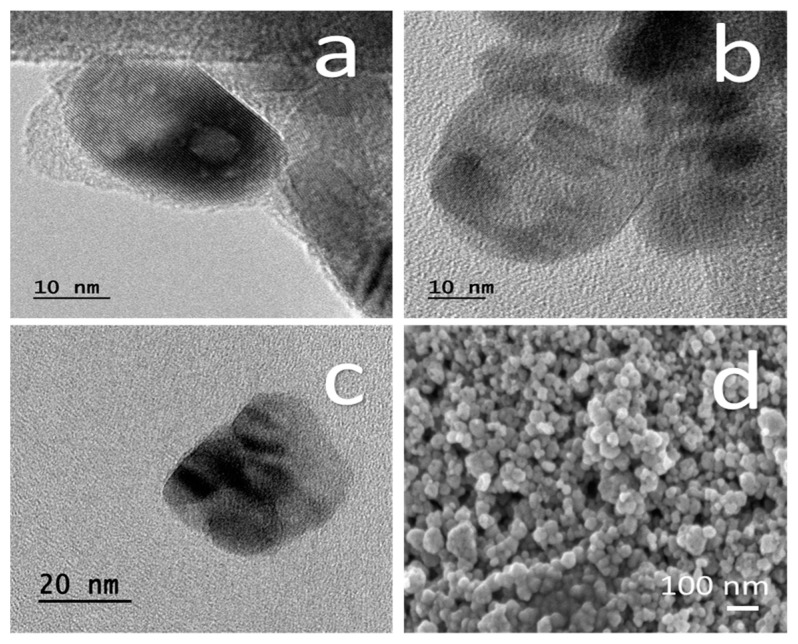
Images of TiO_2_ Nps with TEM in (**a**–**c**) and with SEM is shown in (**d**).

TEM images of TiO_2_ NPs are shown in Figure 3a–c. The average aspect ratio was 5 to 30 nm. All the particles were close to a sphere shape (0.87 ± 0.01 circularity parameter). The SEM images show the size and morphology of the TiO_2_ NPs (Figure 3d). In addition, it shows the agglomerated clusters of NPs.

**Figure 4:**
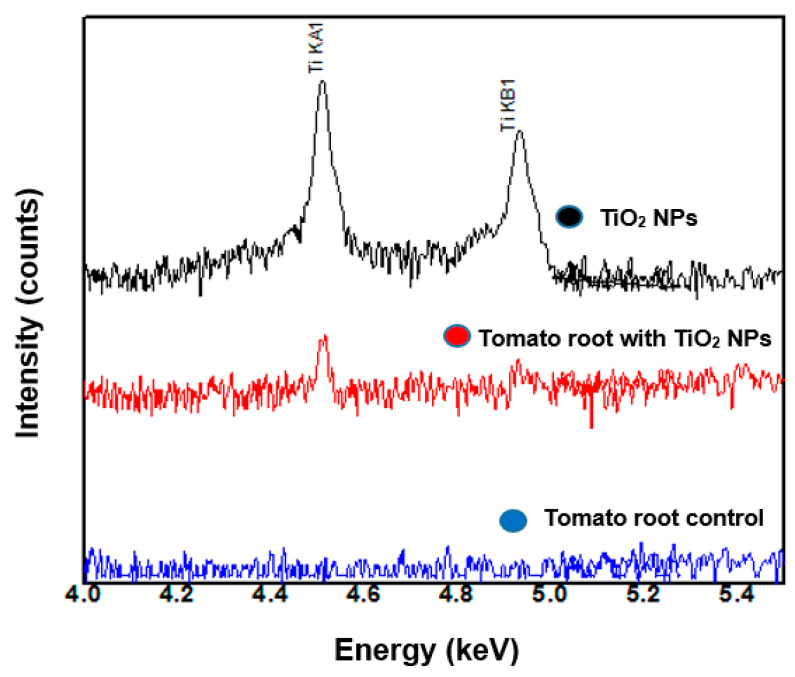
WDXRF analyses of tomato root control (blue line), and the root exposed to 20 mg/L of TiO_2_ NPs (red line) and TiO_2_ NPs (black line).

TiO_2_ NPs were analyzed in WDXRF to generate a control spectrum for Ti, and then measurements in tomato root samples with and without TiO_2_ NPs were incorporated into the MS medium. Figure 4 shows the WDXRF spectrum of TiO_2_ NPs (black line), two peaks corresponding to Ti element at k_α_ on 4.51 and k_β_ at 4.95 keV were observed. With less intensity, the same peaks are observed when we incorporate the nanoparticles into the tomato growth medium (red line). The control spectrum for tomato root does not present any peaks in these energies (blue line). 

### 3.2. Changes in Tomato Root Cell Morphology after Exposure to TiO_2_ NPs

**Figure 5:**
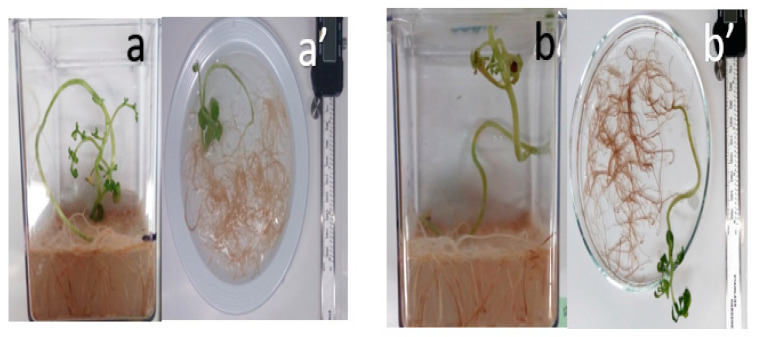
Tomato root plant in Magenta Vessel culture (**a**,**b**) control and exposed to TiO_2_ NPs at 21 days and their root length respectively (**a**’,**b**’).

The changes in tomato root length were recorded. In control samples, the length was 30.14 mm, 47.84% less than the length of the tomato root that was exposed to TiO_2_ NPs. These results could indicate that the TiO_2_ NPs are stimulating the elongation or proliferation of tomato root cells (Figure 5). 

**Figure 6:**
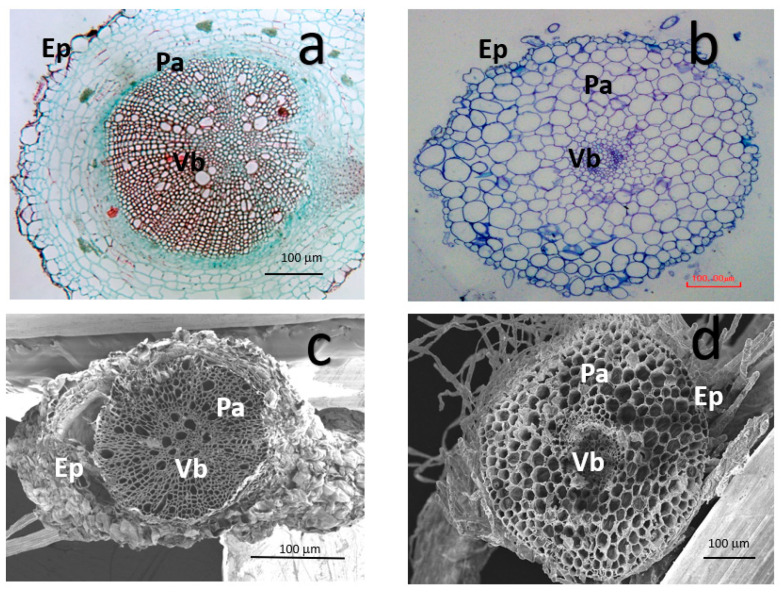
Microstructure characterization of tomato root at 21 days of growth in LM and SEM. Tomato root control (**a**,**c**) and exposed to TiO_2_ NPs (**b**,**d**) with LM and SEM, respectively.

The cells’ microstructure of the tomato root samples characterized by LM in three zones, Ep, Pa and Vb (Figure 6).

**Figure 7:**
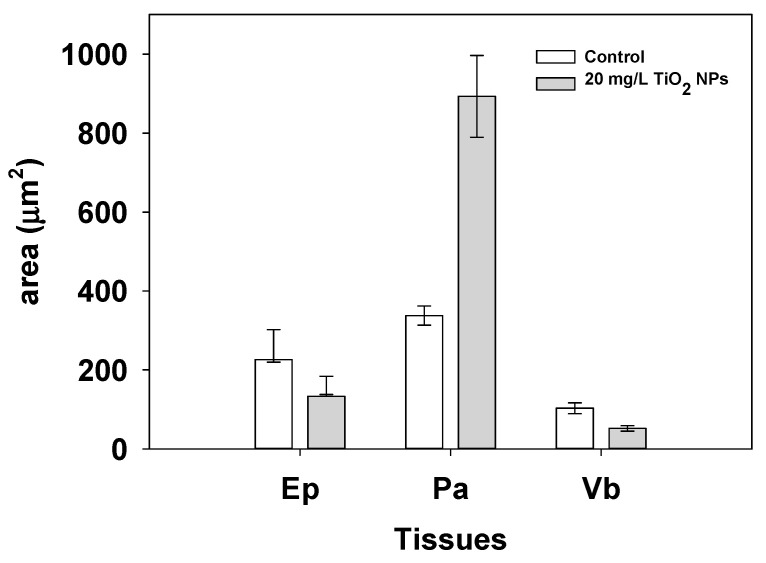
Tissue area of the tomato root control and the root exposed at 20 mg/L of TiO_2_ NPs for Ep, Pa, Vb. Bars indicate standard errors.

The results show that tomato roots exposed to TiO_2_ NPs had a decreased area from 260.92 ± 41.15 µm^2^ to 160.71 ± 22.93 µm^2^ (*p* < 0.05) in Ep and Vb, area changes from 103.08 ± 13.88 µm^2^ to 52.13 ± 7.16 µm^2^. Similarly, the Pa tissue is observed to increase area from 337.72 ± 24.23 to 892.96 ± 463.3 µm^2^ (*p* < 0.05) (*p* < 0.05, Figure 7). 

**Figure 8:**
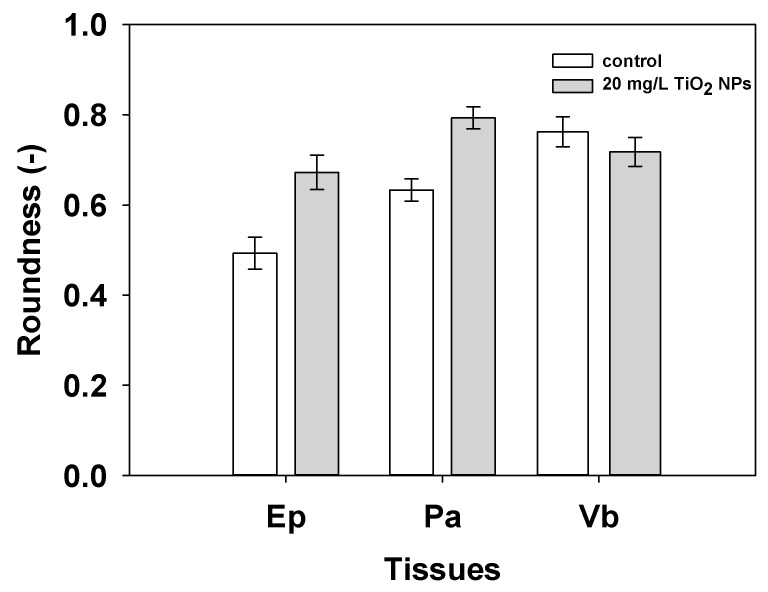
Cellular roundness by tissue type for the tomato root control, and the root exposed at 20 mg/L of TiO_2_ NPs for Ep, Pa, and Vb. Bars indicate standard errors.

In terms of the roundness parameter: when the cell shows a 1.0 value it means that the shape of the cell is perfectly spheric. A vegetal cell does not have this shape in a normal way. It is more flattened in the peripheric zone (Ep) than the Pa and Vb zone. When tomato root is exposed to TiO_2_ NPs some changes in the microstructure are visible (Figure 6). The Ep changed from 0.49 ± 0.03 to 0.67 ± 0.03 µm^2^, the Pa changed from 0.63 ± 0.02 to 0.79 ± 0.02 µm^2^ and the Vb changed from 0.76 ± 0.14 to 0.71 ± 0.03 µm^2^ with incorporation of TiO_2_ NPs. All treatments showed statistical differences (*p* < 0.05, Figure 8).

### 3.3. Nanomechanical Properties

**Figure 9:**
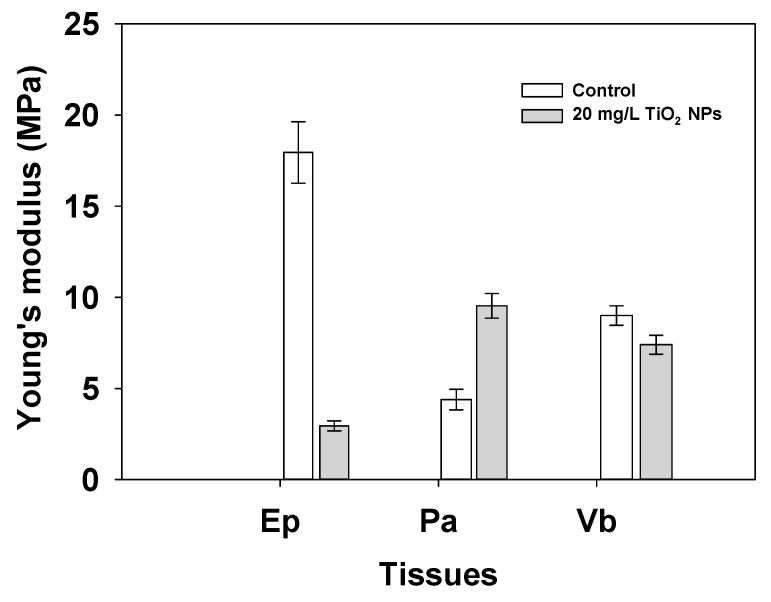
Young’s Modulus of the tomato root control and the root exposed to 20 mg/L of TiO_2_ NPs for Ep, Pa, Vb. Bars show standard error.

The nanomechanical properties were measured by an AFM and showed that the mechanical behavior changes in the tomato root during its growth when exposed to TiO_2_ NPs. The values of *E* in the Ep tissue decreased from 13.9 ± 5.98 to 1.06 ± 0.28 MPa. This suggests that the stiffness decreased when the tomato root was exposed to TiO_2_ NPs. The values of *E* in the Pa tissue increase from 0.48 ± 8.6 to 4.98 ± 0.68 MPa, and *E* in Vb shows a change from 6.37 ± 0.53 to 4.41 ± 0.50 MPa (*p* < 0.05, Figure 9). According to Xi et al. [23], the values of *E* reported in the tomato root are like the values of *E* reported in an onion (Allium cepa) (22.8 MPa). It is possible that the *E* values are smaller due to the anisotropy of the sample.

### 3.4. Localization of TiO_2_ NPs in Tomato Root Cells 

WDXRF was used to measure the concentration of Ti from TiO_2_ NPs incorporated in the tomato root. Figure 4 shows the WDXRF spectrum measurement of the TiO_2_ NPs (black line). Two peaks were detected corresponding to Ti k_α_ at 4.51 and k_β_ at 4.95 keV. These peaks did not appear in the measured spectra taken from the tomato root control sample (blue line); nevertheless, they are clearly observed in tomato root samples exposed to 20 mg/L of TiO_2_ NPs (red line). This indicates that the tomato root does not have naturally occurring titanium. When the tomato root was exposed to TiO_2_ NPs, the root incorporated them. The WDXRF assay confirmed the incorporation of titanium from TiO_2_ into the tomato root.

**Figure 10:**
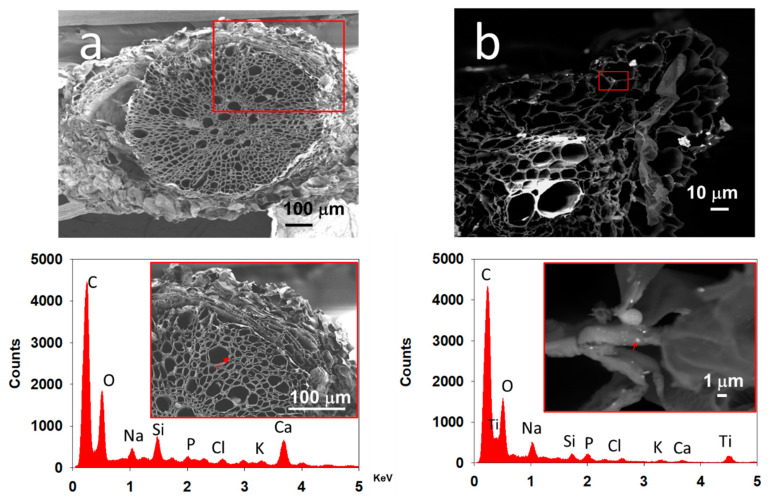
Element detected by SEM/DLS in tomato (**a**) root control and (**b**) exposure at 20 mg/L TiO_2_ NPs.

Tomato root was measure by SEM/EDS to find its chemical composition. In the control sample were detected Carbon (55.6% weight), Oxygen (38.64% weight), Magnesium (1.41% weight), Phosphorous (1.67% weight) y Chloride (2.68% weight), but no Titanium was detected. Nevertheless, tomato roots exposure at 20 mg/L o_f_ TiO_2_ NPs were analyzed in the same way, and the detection of Titanium shows 0.54% weight. Additionally, tomato seeds were tested to the same assay, and the elements detected were Phosphorous (2.50% weight), Potassium (2.21% weight), Calcium (0.99% weight), Sulfur (0.82% weight), Magnesium (0.56% weight), Silicon (0.53% weight), Iron (0.44% weight), Chloride (0.12% weight), Aluminum (978 ppm), Sodium (681 ppm) and Manganese (0.01 ppm). It means that Ti is not found inside of tomato seeds, but it was found in tomato root tissue, which means TiO_2_ NPs were incorporated in the plant system (Figure 10). 

**Figure 11:**
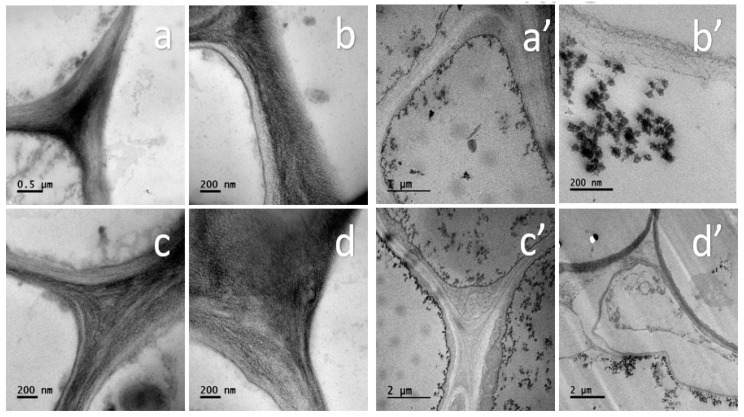
Ultrastructure of tomato root by TEM, with and without TiO_2_ NPs. (**a**–**d**) cell walls without TiO_2_ NPs. (**a**’–**d**’) cell walls with TiO_2_ NPs.

After 21 days of exposure to 20 mg/L TiO_2_ NPs, tomato root cells showed TiO_2_ NPs inside the cells near to cell wall, this could be observed by image analysis of TEM. Figure 11 shows the incorporation of TiO_2_ NPs inside the vegetal cell of the tomato root.

## 4. Discussion

TiO_2_ NPs obtained from the supplier were determined at different size for each technique, like XRD (14 nm average size, Figure 1), DLS (5 to15 nm distribution with 7.5 nm maximum, Figure 2), and TEM (range between 5–30 nm, Figure 3). Each technique has different physical principles, and the tendency of NPs to agglomerate might slightly change the results. In XRD, the sample was also measured in dry powder, and in DLS, the sample was dispersed in an ethylene glycol-water mix (1:3) and sonicated for 20 min. In the TEM test, the TiO_2_ NPs were measured in dry conditions and dispersed in alcohol. These results suggest that TiO_2_ NPs hydrodynamic diameter by DLS is smaller. This method is very similar to the aqueous medium to growth the tomato root. DLS size analyses might be closer to the real behavior of free TiO_2_ NPs in a wild environment.

The incorporation TiO_2_ NPs into the tomato root was determined by WDXRF (Figure 4) and SEM/EDS (Figure 10b) with Ti detection. The results indicate that the tomato root does not have naturally occurring Ti and when the tomato root was exposed to TiO_2_ NPs, the root incorporated them. 

Some studies in root cells tend to not show effects in plants exposed to TiO_2_ NPs, for example, Lu et al. [24] reported no effects in root elongation of corn and rice. Asli and Neumann [25] showed primary corn root is not affected after the exposure to 0.3 and 1 g/L of TiO_2_ NPs (30 nm) during 3 days of treatment. Visually, the roots were not affected, but other organs, like sheets, experienced the opposite effect.

Figure 5 shows the comparison of the length of the tomato root when it is not exposed at TiO_2_ NPs and when it is exposed. The results reveal that the tomato root exposure to TiO_2_ NPs increased in length versus the control root, these results are according to Raliya et al. [8] who reported tomato plants sprayed with TiO_2_ NPs indicated a decrease in root length and, tomato roots exposed to the soil treatment with TiO_2_ NPs showed a root length increase of 250 mg/kg TiO_2_ NPs without any difference at higher concentrations. Even though some species like Allium cepa [7], Zea mays and Lycopersicum esculentum presented smaller or no effect in root length exposed to TiO_2_ NPs. It is not a behavior rule to the vegetable kingdom. Samadi et al. [26] showed that Menta Piperita increased its length root at 1000 mg/L and decreased at 200 mg/L of TiO_2_ NPs exposure. It could be that TiO_2_ NPs promoted the root growth at a determined concentration in M. piperita, but the germination had a reduction without TiO_2_ NPs treatment. Some of these changes could be associated with lignin degradation by TiO_2_ action catalysis. It is known that TiO_2_ has a photocatalytic effect in phenolic components, such as lignin [27]. The TiO_2_ NPs under the light/dark conditions were exposed to light, and the lignin inside of the tomato root could have affected its morphology and mechanical properties.

Morphological changes in the tomato root were detected in the cell area and roundness. Figure 6a,b provides evidence of this change, the cells exposed to TiO_2_ NPs presented a reduction in a Va area, and the shape of the cell tends to be circular instead of quadrangular shape, like in the control root. The areas of tomato root were identified by the shape and looks of the cell wall; Ep show a light layer of cells at the periphery, the Pa looks like a rectangular shape and light cell wall, and Va were identified by its tick cell wall. The cell area per tomato root zone was determined, and the results show a decrease the cell area in Ep and Vb, however, Pa cells had a high increase in their area. With this result, a proposal can be done, that Pa had a growth induced by exposition to TiO_2_ NPs. All tomato root zones presented changes in their shape, but Pa presented the major changes, including the cell roundness value, where the morphological shape of the cells looks like a circular geometry. These changes are associated with the stress response to tomato root exposed to TiO_2_ NPs [18].

On the other hand, *E* shows different mechanical behavior in tomato root zones, in Ep it shows a significant decrease of this value. This means that the stiffness in this zone are lower when it is exposed to TiO_2_ NPs. There are a few reports that contrast the different zones of root and their stiffness value. However, according to Xi et al. [23], the values of *E* reported in the tomato root Ep in this study are similar to the values of *E* reported in Ep of onion (Allium cepa) (22.8 MPa) in normal conditions. It is possible that the *E* values are smaller due to the anisotropy of the sample. The tomato root grows inside of soil and it needs to break soil tension forces to take water and/or nutrients from the soil, instead, the onion is a modified root like a bulb, and their behavior is mainly for storage nutrients and water. Other studies identified the *E* in a different zone of a plant, for example, in Arabidopsis root in the meristematic zone at the center and flanks reported 5 ± 2 and 1.5 ± 0.7 MPa of *E* values, this means that the analog zone of Ep and Vb presented a difference despite both being tissues in the same area, just like this study [21].

Moreover, in Pa tissue, a high increase in *E* values was observed, this indicates that tissue has a mechanical resistance behavior in the presence of TiO_2_ NPs, and the response tends to increase stiffness. Some studies were carried out in the mesocarp of the apple where the *E* showed low values (0.86 ± 0.81 MPa) [19,20]. However, these values may be influenced by the measurement conditions, such as isolated cell or in tissue. The critical changes took place in this zone of the root. When a tomato plant is exposed to TiO_2_ NPs, the Pa change in area, shape cell and stiffness. Some of these effects could be associated with the physiological function of Pa, store water and nutrients. TiO_2_ NPs could be stored in this cell and contribute to its increased stiffness.

In the case of Vb, the values of *E* decreased from 6.37 ± 8.23 to 4.41 ± 0.50 MPa. However, this change does not show a statistical difference (*p* > 0.05, Figure 7). These results mean that Vb tried to maintain the same stiffness with or without TiO_2_ NPs. A meristem apical study of Arabidopsis reported that the values of *E* in the center region are higher (5 ± 2 MPa) than flank zones (1.5 ± 0.7 MPa). These results show the same tendency as the tomato root samples used in this study, where the center corresponds to the Vb and the flanks to the Ep [21]. These changes could be associated with the growth and tension forces that the three tissues experienced during growth, and the resistance that the tomato root felt in the presence of an external change in its medium growth.

In the SEM/EDS and TEM assays, TiO_2_ NPs were detected inside the tomato root cell (Figure 10 and Figure 11). In SEM images with EDS, it was too difficult to determine the sites where the TiO_2_ NPs were, but Ti was found in specific zones of tomato root. TEM analysis inside the cells shows that TiO_2_ NPs are in the form of agglomerates very close to the wall of the cells. (Figure 11).

## 5. Conclusions

Two phases of TiO_2_ NPs were found: anatase (97.4%) and rutile (2.6%) by XRD. The crystallite size average was 14 nm determined without dispersion and, when the TiO_2_ NPs were dispersed in ethylene glycol-water, the average was 5–15 nm determined by DLS. TiO_2_ NPs were agglomerated and need to be dispersed to be incorporated into the MS growth medium.

TiO_2_ NPs were transported into the tomato root during growth into MS medium, and caused morphological changes in area and roundness. The Ep and Vb area decreased, while the Pa increased when TiO_2_ NPs were incorporated. The roundness in the cell was also higher in the Pa, but in the Ep and the Vb it was not affected.

TiO_2_ NPs changed the mechanical properties of the tomato root; Pa became more rigid and the Ep and Vb were softer than the tomato root control. 

In the tomato root control (without NP), Ti was not detected. Meanwhile, the tomato root exposed to 20 mg/L of TiO_2_ NPs contained Ti in WDXRF and SEM-EDS analysis in the tomato root samples.

TEM analysis showed that the TiO_2_ NPs get inside the tomato root cells and are stored in the internal cell space and cell wall, without homogeneous distribution. The TiO_2_ NPs were widely dispersed in all cell spaces.

The effects of TiO_2_ NPs in the tomato roots can be studied in different ways: physiological, morphological, and mechanical behavior. There is a lot of work to be done to know the advantages and/or disadvantages that cause the use of TiO_2_ NPs. The characterization techniques: WDXRF, SEM and TEM confirmed that TiO_2_ NPs were incorporated inside the tomato root. 

This study contributes to the investigation of morphological changes in cells and their stiffness behavior of tomato root in their different zones, Ep, Pa and Vb when is exposed to TiO_2_ NPs. The different effects depend on the tomato root zone. The most relevant change is the increase of *E* in Pa cells associated with an increased area and roundness cell. This zone of the root might be trying to keep the TiO_2_ NPs to avoid their conduction to the Vb to prevent their translocation to another organ, such as leaves or fruit. To the authors’ knowledge, the mechanical properties have not been measured in plant tissues exposed to nanoparticles, particularly to TiO_2_ NPs.

The techniques implemented in this study and the results obtained may help future research into resistant vegetable species under environmental conditions. Stress, translocation of nutrients and nanomaterials in vegetable tissues at the nanoscale level is important for crops.
